# Effect of lipid-bound apolipoprotein A-I cysteine mutant on ATF3 in RAW264.7 cells

**DOI:** 10.1042/BSR20160398

**Published:** 2017-02-03

**Authors:** Yunlong Wang, Yanhui Wang, Shaoyou Jia, Qingzhe Dong, Yuanbin Chen, Shulai Lu, Lin Hou

**Affiliations:** 1 Biological Specimen Bank, The Affiliated Hospital of Qingdao University, Qingdao, China; 2 Department of Biochemistry, Medical College, Qingdao University, Qingdao, Shandong, China; 3 Department of Opthalmology, The Affiliated Hospital of Medical College Qingdao University, 16 Jiangsu Road, Qingdao, China; 4 Department of Stomatology, Qingdao Municipal Hospital, Qingdao, China

**Keywords:** ATF3, inflammatory, lipopolysaccharide, rHDL

## Abstract

Activating transcription factor 3 (ATF3) is a TLR-induced repressor that plays an important role in the inhibition of specific inflammatory signals. We previously constructed recombinant high density lipoproteins (rHDL) (including rHDL_WT_, rHDL_M_, rHDL_228_ and rHDL_74_) and found that rHDL_74_ had a strong anti-inflammatory ability. In the present study, we investigate the roles of recombinant apolipoprotein A-I (ApoA-I) (rHDL_WT_) and its cysteine mutant HDLs (rHDL_M_, rHDL_228_ and rHDL_74_) on ATF3 function in RAW264.7 cells stimulated by lipopolysaccharide. Our results showed that compared with the LPS group, rHDL_74_ can decrease the level of TNF-α and IL-6, whereas rHDL_228_ increases their expression levels. RT-PCR and Western blotting results showed that compared with the LPS group, rHDL_74_, rHDL_WT_ and rHDL_M_ can markedly increase the expression level of ATF3, whereas the level of ATF3 decreases in the rHDL_228_ group. In summary, the different anti-inflammatory mechanisms of the ApoA-I cysteine mutants might be associated with the regulation of ATF3 level.

## Introduction

As a basic and most common indicator of the disease process, the inflammatory response can be defined as the penetration of infectious agents, the enhancement of antigen and the response to cellular injury [1]. In most cases, the inflammatory response is eventually controlled by the release of endogenous anti-inflammatory mediators or by accumulating negative regulatory factors within the immune cell. Such mechanisms allow these inflammatory cells to be cleared at the appropriate time [2]. However, these mechanisms of negative regulation, including the persistent accumulation of negative regulatory factors and activation of white blood cells, may become dysfunctional and, thus, uncontrollable [3]. Epidemiological studies have shown that HDL or its component apolipoprotein A-I (ApoA-I), plays a significant role in anti-inflammatory and antioxidative activities [4,5] and is vital in reverse cholesterol transport [6].

Macrophages are one of the most important immune cells *in vivo* [7]. Macrophages play a crucial role in the immune response because they can kill a pathogen either directly through phagocytosis or indirectly through the secretion of a series of anti-inflammatory mediators [8]. Inflammatory mediators generated by activation of macrophages are associated with several pathophysiological diseases [9], such as rheumatoid arthritis and pulmonary fibrosis [8]. LPS is the main component of the cell wall of Gram-negative bacilli and is a main component of the inflammatory reaction [10]. The inflammatory reaction induced by LPS could induce the expression of inflammatory cytokines through a series of signal transduction, involving transcription factor activation after combining with the LPS receptor CD14 and TLR4 [11]. LPS-stimulated macrophages can serve as a model for studying inflammation and anti-inflammatory diseases [12,13]. In recent years, many animal studies have found that HDL can chelate LPS to inhibit the inflammatory response by preventing activation of intracellular TLR4 [14]. Activating transcription factor 3 (ATF3) is a negative regulator of a particular set of TLR4-induced pro-inflammatory cytokine genes (e.g. *TNF, IL-6* and *IL-12p40*) [15]. ATF3 expression is induced by TLR stimulation or various other stimuli [16] and operates by a negative feedback system to limit the overproduction of pro-inflammatory cytokines such as IL-6, TNF-α and CD14 [17,18]. Furthermore, studies have shown that HDL mediates the anti-inflammatory transcriptional reprogramming of macrophages via the transcriptional repressor ATF3 [14]. HDL can antagonize TLR responses by regulating ATF3 expression both *in vitro* and *in vivo*, which demonstrates that HDL plays a critical role in anti-inflammatory activity [14]. Furthermore, our current understanding of the role of ATF3 in innate immune cells is that ATF3 is a vital inducible repressor of specific transcriptional networks within the innate inflammatory response [19].

In previous studies, we constructed rHDLs (rHDL_WT_, rHDL_M_, rHDL_74_ and rHDL_228_) by combining wild-type or mutant ApoA-I in a solution of 1,2-dipalmitoyl-sn-glycero-3-phosphocholine (DPPC), and found that they have different anti-inflammatory properties [20,21]. In the present study, we used an LPS-induced inflammatory response in RAW264.7 cells to investigate whether the anti-inflammatory properties of these rHDLs are related to the regulation of ATF3 expression.

## Materials and methods

### Reagents and antibodies

LPS and 1,2-dipalmitoyl-*sn*-glycero-3-phosphocholine (DPPC) were purchased from Sigma. ELISA kits were purchased from BlueGene. The Pierce BCA Protein Assay kit and Detoxi-Gel™ Endotoxin Removing Gel were purchased from Thermo. TRIzol Reagent was purchased from Life Technologies. Ni-NTA His Bind resin was purchased from Novagen. PrimeScript^™^ RT reagent Kit with gDNA Eraser and SYBR Premix Ex Taq were purchased from Takara. High-glucose DMEM was purchased from HyClone. FBS was purchased from Gibco. The following antibodies were used: anti-ATF3 and secondary antibodies (anti-rabbit IgG (whole molecule)-peroxidase produced in goat (Sigma; 1:8000), monoclonal GAPDH (Sigma; 1:5000) and secondary antibodies (anti-mouse IgG (Fc specific)-peroxidase produced in goat (Sigma; 1:14000).

### Cells

RAW264.7 mouse macrophages were purchased from the Shanghai Cell Bank. Recombinant *Escherichia coli* containing the coding region for human ApoA-I and cysteine mutant was preserved in the Laboratory of The Affiliated Hospital of Qingdao University. All animal experiments were approved by the animal care committee of the Affiliated Hospital of Qingdao University.

### Extraction and purification of recombinant apolipoproteins

The expression and purification of recombinant ApoA-I and cysteine mutants were based on previous methods, except for the following alterations. The recombinant ApoA-I and cysteine mutants were purified by nickel column chromatography and concentrated using enrichment centrifuge tubes. Proteins were then resolved by SDS/PAGE and quantified using BCA kit. Removal of endotoxin and construction of rHDLs were carried out as described previously [18,19]. Purified proteins were stored at –20°C.

### Cell culture and pretreatment

RAW264.7 cells were cultured in DMEM supplemented with 10% heat-inactivated FBS, 100-units/ml penicillin and 100 μg/ml streptomycin at 37°C in an incubator with humidified air and 5% CO_2_. Cells were treated with trypsin and centrifuged at 1500 rev/min for 5 min for passaging. Cells were cultured in six-well plates (5 × 10^5^ cells per well).

The RAW264.7 cells used in our study were divided into a total of six groups (control, LPS groups and four test groups: rHDL_228_, rHDL_WT_, rHDL_M_ and rHDL_74_). All groups were cultured in 2-ml complete medium for 20 h; the medium was replaced and 1 μg/ml LPS was added to all groups, except the control group, followed by culture for 24 h. Then, 500 μg/ml of the different rHDLs were added into the corresponding test groups for 10 h. Morphological changes were observed by microscopy.

To investigate the cytotoxic effect of different rHDLs, cell viability was measured with the Cell Counting Kit-8 according to the manufacturer’s protocol. Briefly, 1 × 10^3^ cells/well were seeded in 96-well plates, then treated with 3.125, 6.25, 12.5, 25 or 50 μg/ml different rHDLs for 24 h, after that the culture medium was replaced with 100 μl of medium containing 10 μl of CCK-8 per well and the cells were incubated for one more hour. Then, the cell viability was measured.

### Measurement of cytokines expression by ELISA kits

The production of cytokines IL-6 and TNF-α in cell-culture supernatants were measured by ELISA using the manufacturer’s instructions.

### RNA isolation and SYBR Green qPCR

Total RNA was isolated using TRIzol (Life Technologies) according to the manufacturer’s instructions, and the concentration of total RNA was assessed using NanoDrop 2000C (Thermo, U.S.A.). Approximately, 1 μg of extracted RNA from each sample was transcribed to cDNA using a PrimeScript^™^ RT reagent Kit with gDNA Eraser. cDNA amplification was measured by quantitative real-time PCR using SYBR^®^ Premix Ex Taq II (Tli RNaseH Plus). Primers were purchased from Invitrogen. Murine HPRT: forward 5′-TGAAGTACTCATTATAGTCAAGGGCA-3′ and reverse 5′-CTGGTGAAAAGGACCTCTCG-3′. Murine ATF3: forward 5′-GACTGAGATTCGCCATCCA-3′ and reverse 5′-CCGCCTCCTTTTCCTCTCAT-3′. Primer specificity was checked by melt curve analysis. Each treatment includes three replicates with the SYBR green mix in the CFX96 (Bio–Rad Laboratories, Hercules, CA). The expression of the target gene was normalized to the house-keeping gene *HPRT*. Relative quantification of gene expression was performed using the difference in threshold cycle (*C*_T_) method (Δ*C*_T_ = *C*_T_ target –* C*_T_ control) and the relative expression equaled 2^−ΔΔ*C*^_T_ (ΔΔ*C*_T_ = Δ*C*_T_ target – Δ*C*_T_ control). All data are presented as fold change relative to the control.

### Western blotting for ATF3

To confirm *ATF3* mRNA translation in cells, we used Western blotting analysis. Proteins were extracted from the cells, and the protein concentration was measured using a BCA assay kit. Equal amounts of protein per sample were applied to pre-cast SDS/PAGE (12% gels) with MOP buffer and proteins were transferred to PVDF membranes. Membranes were incubated with 5% BSA in Tris buffer for 2 h at room temperature and overnight at 4°C with specific primary antibody. Then, membranes were washed with TBST, incubated with secondary antibodies for 1 h and washed for more than three times. Immunoreactivity was visualized, and blots were scanned for analysis using the Image J2x software.

## Statistical analysis

Values were shown as mean ± S.D., and differences between groups were analysed using one-way ANOVA analysis using SPSS 21.0 software for Windows (SPSS Inc., U.S.A.). *P*<0.05 was considered statistically significant. Additional analyses and making graphs were performed with Prism 6.0 (GraphPad Software Inc.).

## Results

### Expression and purification of rHDLs

**Figure 1:**
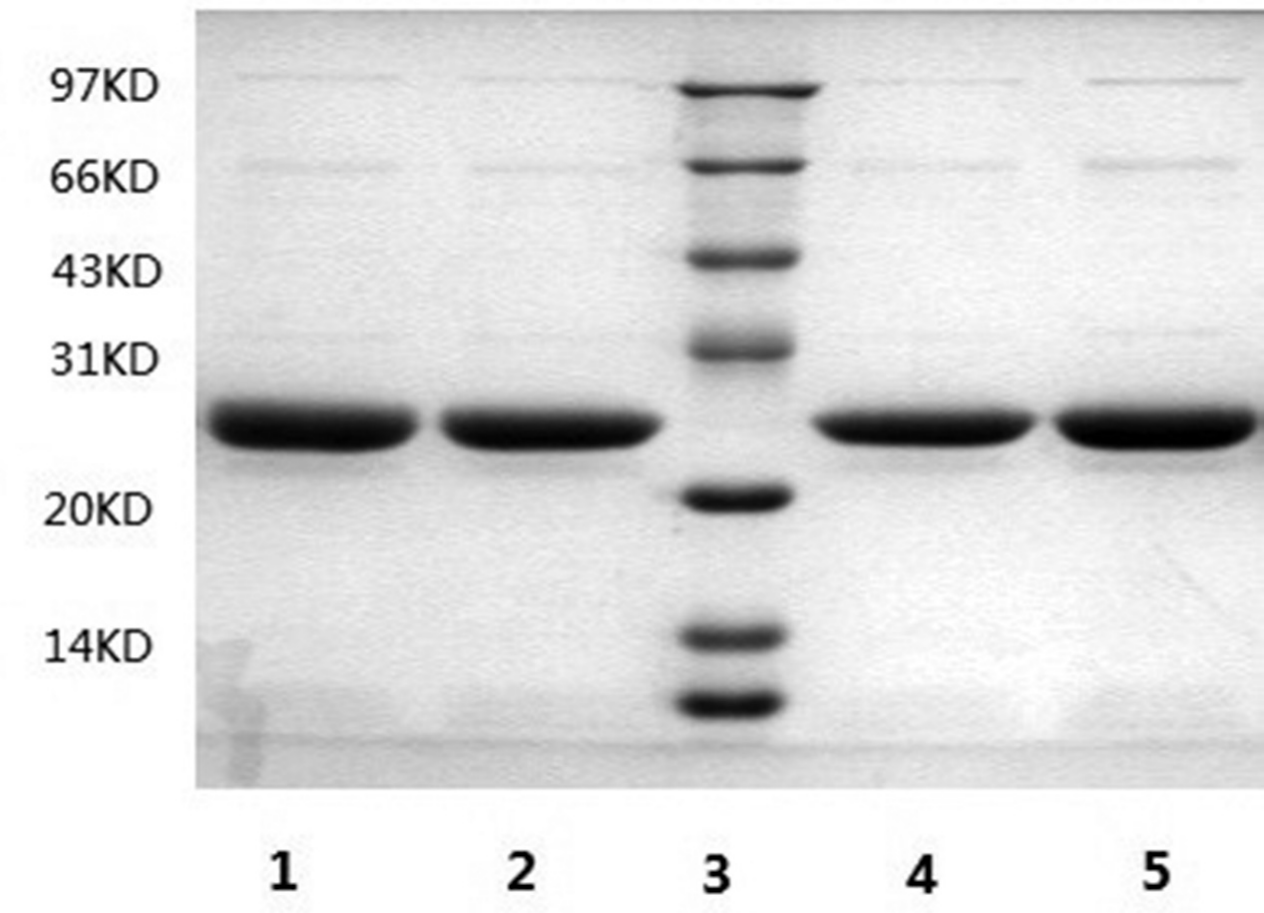
Recombinant purified ApoA-I and its mutants were examined by SDS/PAGE (12% gel) Lanes 1–5 represent wild-type ApoA-I, A-I (N_74_C), low molecular mass marker, A-I (R_173_C) and A-I (S_228_C).

The recombinant plasmid was transferred into *E. coli* and cultured in LB medium. Then, *E. coli* was collected and disrupted for purification of proteins on an Ni^2+^ affinity column, followed by SDS/PAGE (12% protein gel). Coomassie Brilliant Blue staining was used to visualize the 28-kDa protein of interest present in the lysate (Figure 1). These data implied that the protein of interest can be constructed following DPPC embedded experiments to make rHDL.

### rHDLs at low concentrations had no cytotoxic effect on RAW264.7 cells

**Figure 2:**
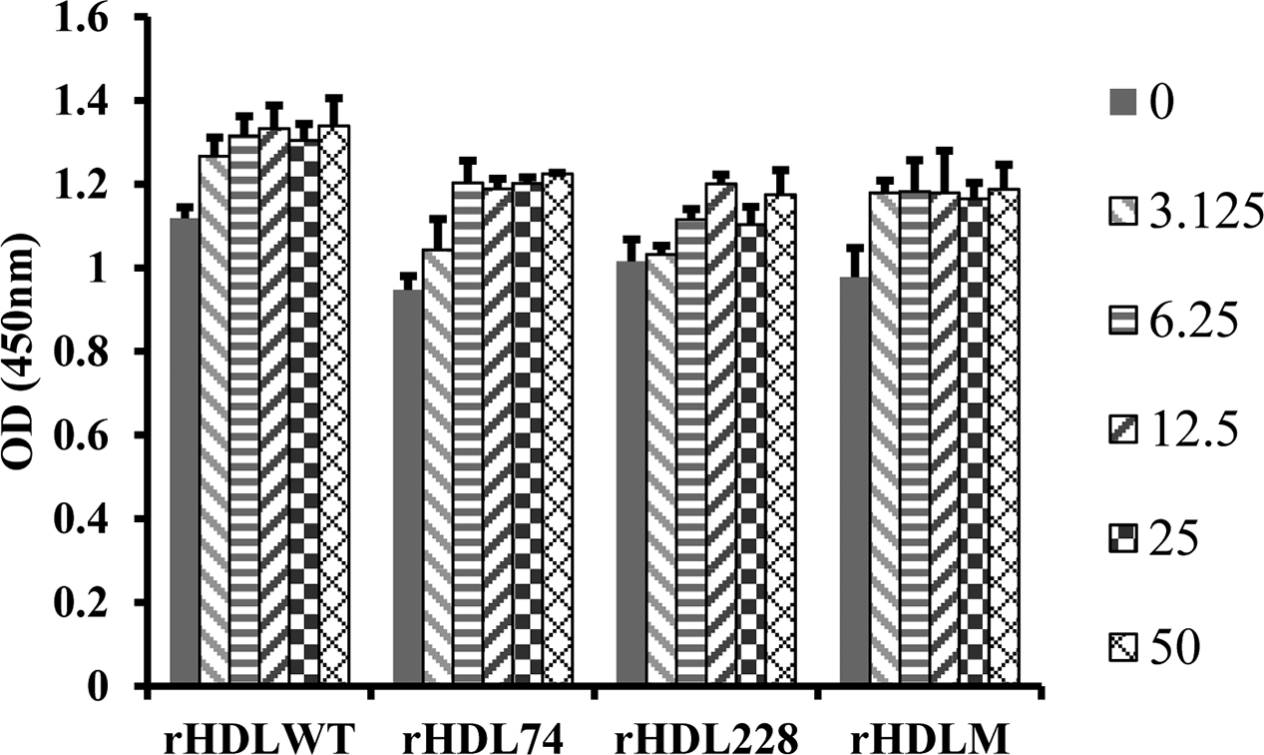
CCK-8 assay after treating with different rHDLs The different columns represent the viability of cells treated with corresponding concentrations of rHDL as shown on the right (μg/ml).

To investigate the effect of rHDLs on cell proliferation and viability, the cells were treated with 3.125, 6.25, 12.5, 25 or 50 μg/ml of different rHDLs for 24 h. The CCK-8 assay showed that rHDLs displayed no cytotoxic effect at a low concentration on RAW264.7 cells (Figure 2).

### Effect of rHDL on the morphological changes of RAW264.7 cells stimulated by LPS

**Figure 3:**
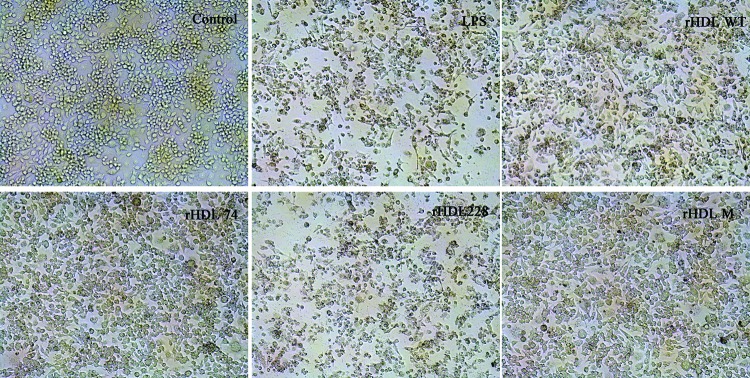
Photomicrographs of representative morphological changes of LPS-induced RAW264.7 cells (SP ×100) Control: without any treatment, LPS, rHDL_WT_, rHDL_74_, rHDL_228_ and rHDL_M_ represent the groups that were treated by LPS, LPS + rHDL_WT_, LPS + rHDL_74_, LPS + rHDL_228_ and rHDL_M_ respectively.

Compared with the control group, the morphology of LPS-treated cells changed obviously (Figure 3): they became diamond-shaped and had pseudopodia and degradation appeared in most cells. The cells in the rHDL_74_ group were significantly restored to a wild-type appearance and were characterized by a spindle or circular shape and showed less degradation compared with LPS group. rHDL_M_ and rHDL_WT_ also had obvious inhibition against inflammation, whereas cells treated with rHDL_228_ had no differences compared with the LPS group.

### Effect of rHDL on the expression of TNF-α and IL-6

**Figure 4:**
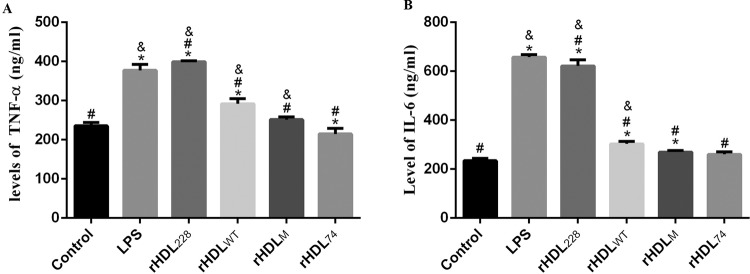
TNF-α and IL-6 levels at 24 h after LPS treatment Compared with the LPS group, the rHDL_74_-treated group showed significantly reduced levels of TNF-α and IL-6. However, rHDL_228_ exhibited an increase for these factors. *P<0.05, compared with control group; ^#^*P*<0.05, compared with LPS group; ^&^*P*<0.05, compared with rHDL_74_ group. (**A**) TNF-α level at 24 h after LPS treatment. (**B**) IL-6 level at 24 h after LPS treatment .

To detect the effect of the rHDLs on LPS-treated cells, we determined the cell culture fluid levels of TNF-α and IL-6 by ELISA. Figure 4A shows that rHDL_WT_ and rHDL_M_ can directly lower the supernatant inflammatory cytokine TNF-α (rHDL_WT_: 291.86 ± 12.77 ng/ml, rHDL_M_: 251.319 ± 7.22 ng/ml, *P*<0.001) compared with the LPS group (377.43 ± 8.09 ng/ml). Compared with the rHDL_WT_, the supernatant level of TNF-α in the rHDL_74_ group (rHDL_74_: 214.77 ± 14.68 ng/ml, *P*<0.01 compared with rHDL_WT_) was significantly reduced. However, the rHDL_228_ (399.366 ± 2.23 ng/ml, *P*<0.001 compared with control) had a much higher level of TNF-α compared with the control group. As shown in Figure 4B, rHDL_WT_ and rHDL_M_ can directly lower the supernatant inflammatory cytokine IL-6 (rHDL_WT_: 303.15 ± 9.70 ng/ml, rHDL_M_: 269.81 ± 6.37 ng/ml, *P*<0.001 compared with LPS) compared with the LPS group (657.83 ± 10.3 ng/ml). Compared with rHDL_WT_, the supernatant level of IL-6 in the rHDL_74_ group (rHDL_74_: 260.264 ± 10.07 ng/ml, *P*=0.0104 compared with rHDL_WT_) was significantly decreased. The level of IL-6 with rHDL_74_ was close to that of the control group (control: 234.647 ± 9.27, *P*=0.0669).

### Effects of rHDLs on the expression of ATF3

**Figure 5:**
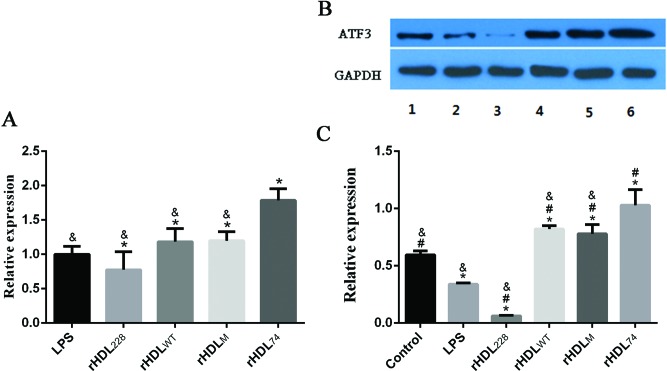
The relative expression of *ATF3* mRNA and protein in LPS-induced RAW264.7 cells (**A**) *ATF3* mRNA relative expression; (**B**) Western blotting analysis after rHDL treatment; (**C**) The grey value analysis of Western blot bands with Image J2x after rHDL treatment. *P<0.05, compared with control group; ^#^*P*<0.05, compared with LPS group; ^&^*P*<0.05, compared with rHDL_74_ group. Error bars indicate S.D. Lanes 1–6 represent control, LPS, rHDL_228_, rHDL_WT_, rHDL_M_ and rHDL_74_.

The anti-inflammatory function of HDLs is closely related to ATF3 expression levels. In animal experiments, HDL injection induced high levels of ATF3 expression [14]. To determine whether ATF3 is responsible for the anti-inflammatory effects of rHDLs, we detected the mRNA levels of ATF3 using quantitative real-time RT-PCR (Figure 5A) and examined the protein levels through Western blotting (Figure 5B, C). Fluorescence RT-PCR showed that compared with the LPS group, the expression level of ATF3 in rHDL_74_ group was the highest; rHDL_WT_ and rHDL_M_ groups also had increased ATF3 expression levels (*P*<0.05). In contrast with other rHDLs, the ATF3 expression level of rHDL_228_ group was lower than the LPS group. The mRNA expression results were consistent with the results of ELISA and cell morphology analysis, which showed that rHDL_74_, rHDL_M_ and rHDL_WT_ can reduce the level of inflammation and enhance the expression level of ATF3. To verify whether enhancement occurred at the translation level to increase protein expression, Western blotting was conducted. Compared with the control group, the expression levels of ATF3 in the LPS group and rHDL_228_ group were significantly reduced and that of the rHDL_228_ group was lower than the LPS group. The expression levels of rHDL_WT_ and rHDL_M_ were increased, and this difference was significant, with the highest expression level was of rHDL_74_. The anti-inflammatory effect of the mutant was most significant for rHDL_74_.

## Discussion

Inflammation is a specific mechanism underlying multiple physiological and disease processes [22]. Studies have shown that HDL can inhibit the development and progression of inflammation and antioxidant effects [23]. The key characteristic in the early phase of inflammation in plaques is the involvement of the innate immune system with respect to the macrophage. These cells are activated in the blood vessel wall in response to lipoproteins containing apolipoprotein B, such as low-density lipoproteins (LDL and VLDL) [24]. The cholesterol carried by HDL causes the macrophages to become ‘foam cells’ and inflammatory cytokines are then secreted by macrophage foam cells [14,25,26]. Studies have shown that HDL in macrophages may reduce inflammation, dependent on the transcription factor ATF3, to activate the anti-inflammatory pathway [27]. In our study, we assessed the anti-inflammatory function of ApoA-I cysteine mutants, and our results showed that the rHDL74 mutant can increase the ATF3 level and exhibit a high anti-inflammatory ability.

ApoA-I is the main component of the HDL, and studies have shown that it plays a major role in cholesterol efflux and anti-atherosclerosis [6,28]. SRC–HDL, CSL-111, CSL-112 and ETC-216 whose main component is ApoA-I or its natural cysteine mutant ApoA-I_M_ had been used in clinical trials. Previous studies have shown that these HDLs can quickly transport cholesterol and eliminate atherosclerotic plaques [6,29–32]. The drug ETC-216 based on ApoA-I_M_ was developed by the Esperion Pharmaceutical Company, and clinical research has shown that the drug has a significant anti-atherosclerosis effect [32]. Previously, we have designed and constructed seven cysteine mutants of ApoA-I containing a natural mutant ApoA-I_M_ i.e. A-I (S_52_C), A-I (N_74_C), A-I (K_107_C), A-I (G_129_C), A-IM (R_173_C), A-I (K_195_C), A-I (S_228_C), and their functions were studied. The 74 and 52 mutants A-I (N_74_C) and A-I (S_52_C) and rHDLs: rHDL_74_ and rHDL_52_ had significantly increased anti-inflammatory functions compared with wild-type, whereas the 228 mutant rHDL_228_ exacerbated inflammation [14,20,21,33]. The mechanism of anti-inflammatory action is not clear.

We, therefore, investigated the effects of rHDLs on LPS-stimulated RAW264.7 macrophages and studied the anti-inflammatory abilities of the rHDL. Our study suggested that high-density lipoprotein (HDL) exerted different effects on inflammatory cytokine expression. Cell morphology and ELISA detection showed that rHDL_74_ had the strongest inhibition of inflammation. Wild-type rHDL_WT_ and rHDL_M_ also inhibited inflammation, but the effect was less than that of rHDL_74_. Correspondingly, to verify the relationship between expression levels of inflammatory factors and rHDL and ATF3 expression, we observed that the anti-inflammatory effect of rHDL was proportional to the expression level of ATF3. rHDL_74_ had the strongest inhibition of inflammation and the expression level of ATF3 was also the highest. At the same time, we observed that rHDL_228_ might have pro-inflammatory effects and we found that the expression level of ATF3 protein and mRNA was the lowest.

ATF3 is the key transcriptional repressor. It can be induced by TLR stimulation and acts by a negative-feedback mechanism to limit excessive production of pro-inflammatory cytokines including TNF, IL-6 and IL-12p40 [17,18]. Although, it is known that HDL can sequester LPS and thereby prevent cellular activation through TLR4 [34], recent evidence indicates that HDL could promote ATF3 expression, leading to down-regulation of TLR-induced inflammatory responses [14]. In the present study, we found that the rHDL_74_, rHDL_WT_ and rHDL_M_ mutants could enhance the expression levels of the TLR ATF3 response to negatively regulate inflammation, whereas rHDL_228_ may inhibit ATF3 activity and aggravate inflammation. As amino acid mutations may affect protein conformation and cysteine residues can be engaged in disulfide bridges, these artificial single amino acid mutations may play their unique roles in the formation of ApoA-I homodimers, thereby influencing the HDL protein function and HDL-ATF3-TLR pathway. In conclusion, the anti-inflammatory function of rHDL that we constructed was positively correlated with ATF3 expression. How these mechanisms develop in different cell types *in vivo* and in different microenvironments is the next major focus of our research and for discerning the relationship between HDL and ATF3 and innate immune research in general.

## Abbreviations

ApoA-I: apolipoprotein A-I
ATF3: activating transcription factor 3
CCK-8: cell counting kit-8
*C*_T_: threshold-cycle method
HDL: high-density lipoprotein
rHDL: recombinant HDL
LPS: lipopolysaccharide
TLR: toll-like receptor
VLDL: very low density lipoprotein
